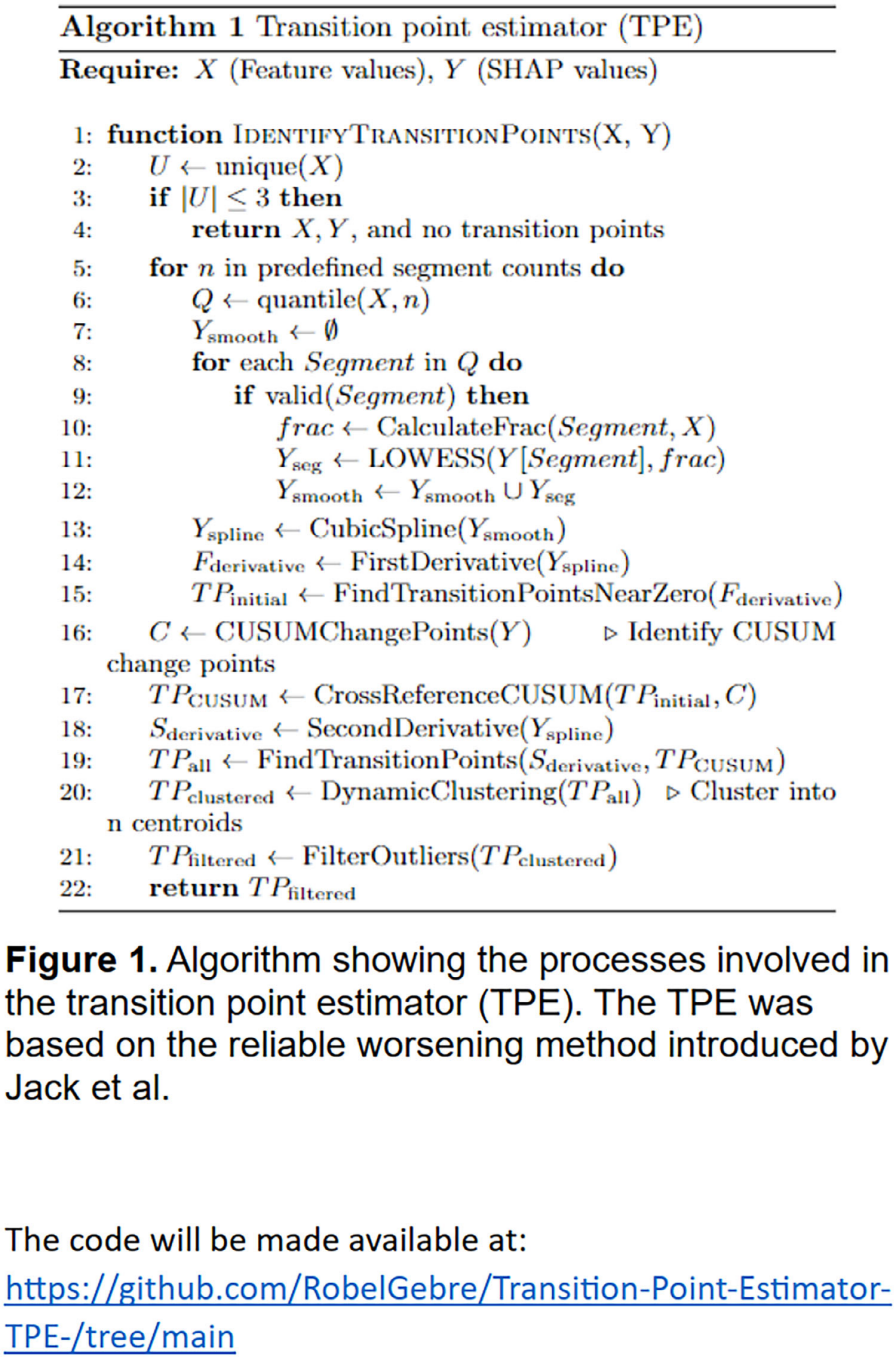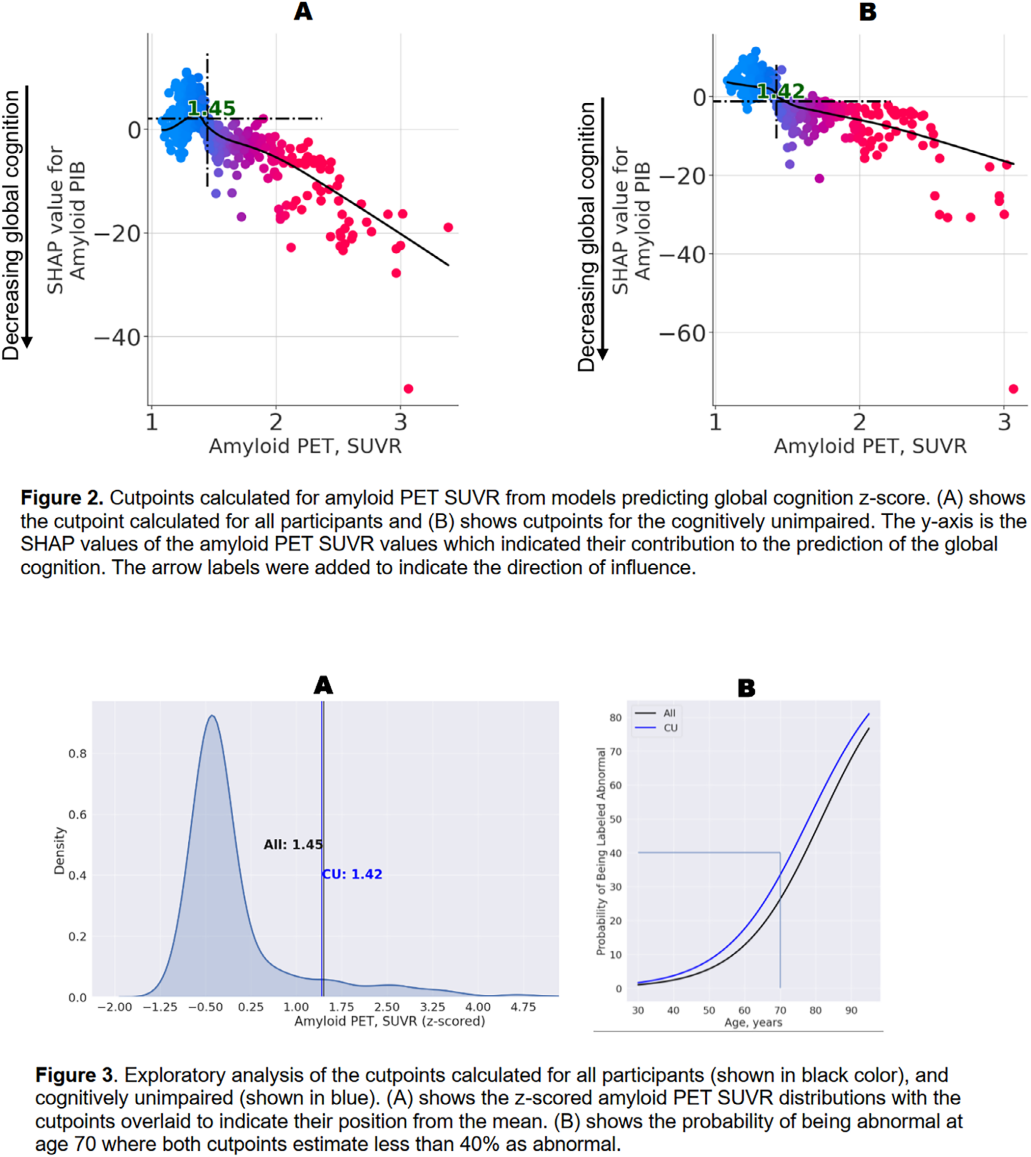# Identifying transition points in AD biomarkers using machine learning: A supplement to the reliable worsening method

**DOI:** 10.1002/alz.090697

**Published:** 2025-01-03

**Authors:** Robel K Gebre, Clifford R. Jack, Prashanthi Vemuri

**Affiliations:** ^1^ Mayo Clinic, Rochester, MN USA

## Abstract

**Background:**

Biomarker cutpoints have received significant importance in AD research. Reliable worsening method based on serial biomarker data has been proposed (Jack et al., 2017 Alz & Dem). Advanced machine learning models (ML) and explainable AI provide an opportunity to enhance existing techniques. We aimed to estimate biomarker cutpoints on cross‐sectional data using ML by identifying important transitions in the biomarker values.

**Method:**

We first trained an ML model with the biomarker of interest as an input including other available biomarkers, demographics, and diagnosis, and predicted global cognition z‐score. We then obtained SHAP values (Shapley Additive exPlanations), fit a curve between the SHAP values and biomarker values, and calculated the first derivatives (slope) of the curve. Then, a CUSUM (Cumulative Sum) analysis was used to identify SHAP change points and cross‐referenced to values where the slopes were steepest near the zero‐crossing location of the curve. A zero SHAP is an important indicator of a feature’s progressive importance for model prediction because a feature’s influence on prediction significantly shifts when its SHAP values go from positive to negative or vice versa. However, relying on zero‐crossing alone may be insufficient when the relationship is non‐linear. Consequently, we calculated the second derivatives to identify inflection points (peaks and troughs) along the curve. Lastly, we compiled all the potential cutpoints and performed post‐processing as shown in Fig. 1.

**Result:**

The algorithm identified cutpoints at 1.44 SUVR for all and at 1.42 SUVR for CU participants (Fig. 2). At age 70, both cutpoints estimated the percentage of abnormals at less than 40% (Fig. 3).

**Conclusion:**

TPE amyloid‐PET SUVR cutpoints for CUs equal the reported RW values (1.42 SUVR). A strength of the TPE is its application on cross‐sectional data making it versatile for various biomarkers across different cohorts.